# Impact of zinc on arbuscular mycorrhizal-mediated nutrient acquisition in urban horticulture

**DOI:** 10.1016/j.isci.2024.110580

**Published:** 2024-07-25

**Authors:** Miles P.A. Bate-Weldon, Jill L. Edmondson, Katie J. Field

**Affiliations:** 1 Plants, Photosynthesis and Soil, School of Biosciences, University of Sheffield, Sheffield S10 2TN, UK

**Keywords:** Mycology, Plant biology, Horticulture

## Abstract

A major barrier to sustainably improving food security for a growing global population is the availability of suitable space for growing crops. Urban areas offer a potential solution to increase availability of land, however, horticultural soils often accumulate zinc. These increased levels may affect the interactions between crops and soil microbes with potential implications for crop health and nutrition. Using radio-isotope tracing, we investigated the effect of urban environmentally relevant concentrations of zinc in soils on the nutrient exchange between arbuscular mycorrhizal fungi and pea plants. At higher concentrations of zinc, transfer of phosphorus from fungi to plants and the movement of carbon from plants to fungi was dramatically decreased. Our results suggest that while urban horticulture holds promise for sustainably enhancing local food production and addressing global food security, the unchecked presence of contaminants in these soils may pose a critical hurdle to realizing the potential of urban soils.

## Highlights

• High concentrations of zinc (Zn), often found in urban soil, reduces pea plant biomass
• High Zn reduced arbuscular mycorrhizal (AM) root colonization but not hyphal density
• AM phosphorus transfer to plants and reciprocal carbon transfer is reduced at high Zn
• Soil Zn may reduce the effectiveness of beneficial microbes in urban horticulture

## Graphical abstract

**Figure:**
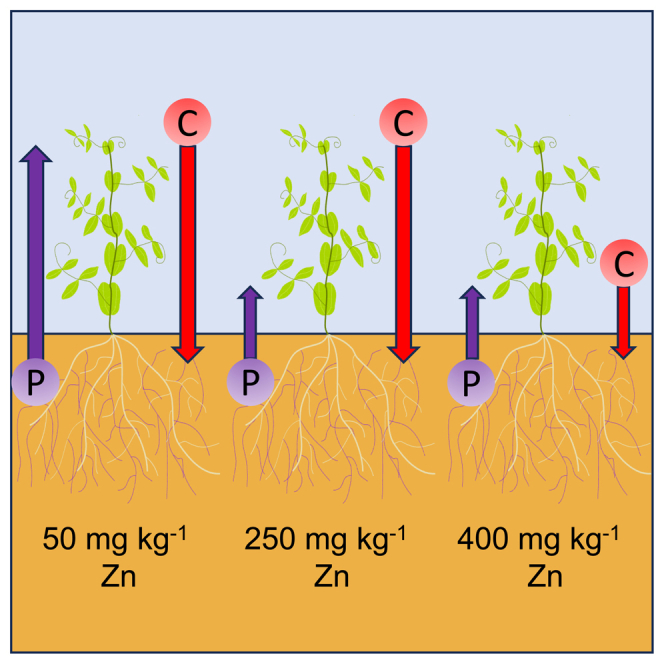

## Introduction

By 2050, 70% of the growing global population^1^ are expected to live in cities and other urban areas,^2^ necessitating a corresponding increase in secure food production of ∼70%.^3^ However, a major barrier to producing more food is the availability of land of appropriate quality on which to grow food, with current land management practices for intensive agriculture enhancing soil degradation and nutrient depletion in areas currently used for food production.^4^^,^^5^ Urban areas are largely reliant on imported food and are therefore vulnerable to supply disruption, often because of global events such as the COVID-19 pandemic and climate change.^6^ This is particularly acute with fresh products such as fruits and vegetables. Urban horticulture currently makes a relatively small contribution to food provision in towns and cities, however, the production of fruit and vegetable crops within and around cities has the potential to increase sustainably if available space in urban areas is repurposed toward food production.^7^^,^^8^

Despite the potential of urban greenspaces to contribute to local food security, there are a number of barriers to increasing food production within cities. These include lack of understanding on how the distinct properties of urban soils may affect the growth of food crops and their nutritional value.^9^ Soils in urban environments, including soils used for urban horticulture, often contain relatively high concentrations of pollutants such as heavy metals, microplastics and black carbon from various anthropogenic sources including modern and historical transport and industry.^9^^,^^10^^,^^11^^,^^12^^,^^13^^,^^14^^,^^15^ However, urban soils used for horticulture can also accumulate heavy metals such as zinc (Zn) as a result of land management practices such as unregulated application of manures and pesticides.^16^^,^^17^ This has resulted in accumulation of heavy metals, including Zn, in urban horticultural soils across the UK.^9^ At high concentrations Zn in the soil can become phytotoxic,^18^^,^^19^^,^^20^ inhibiting key plant processes such as photosynthesis and nutrient uptake,^20^^,^^21^ potentially also impacting crop yields and nutritional values. The concentration in the soil at which Zn starts having an impact on plants depends on a number of factors, such as soil pH, which may affect uptake into the plant tissues. In a study investigating the spatial distribution of Zn in European soils it was found any soils exceeding 167 mg kg^−1^ were in the top 1% of soils sampled with the mean concentration of Zn in topsoil found to be 47 mg kg^−1^.^22^ However, in a separate study, soils used for urban horticulture substantially surpass these values with a national range of 46.16–1213.0 mg kg^−1^, indicating that the prevalence of Zn in urban horticultural soils is a near ubiquitous issue.^9^ Although the risk to human health from consuming food produced on urban soils with elevated heavy metal concentrations is low^9^^,^^12^ , the wider impacts of heavy metal contamination of soil on ecosystem services, particularly those relevant to the urban horticultural sector, such as soil health and functionality, are under investigated.

Soil microbes play critical roles in maintaining soil quality and nutrient cycling and, as such, are critical to the sustainable production of nutritious food crops.^23^^,^^24^ Arbuscular mycorrhizal (AM) fungi are a near-ubiquitous group of soil microbes that interact directly with the roots and root-like structures of most plants^25^ in nearly every major terrestrial biome^26^^,^^27^ including urban environments.^28^ Once within roots of a suitable host plant, fine filamentous extraradical fungal hyphae grow out into soil where they scavenge and assimilate nutrients from the soil in regions that are beyond the reach of the plant roots or those that are otherwise inaccessible such as from tiny soil pores or from soil minerals.^29^^,^^30^ AM fungi transfer nutrients scavenged from the soil to the host plant via intracellular structures within the plant root cortex and, in return, receives an allocation of plant-fixed carbon as carbohydrates and/or lipids.^29^^,^^30^ In this way AM fungi play an important role in plant phosphorus (P) nutrition as well as for plant assimilation of other key nutrients such as nitrogen, a variety of micronutrients, and water.^29^^,^^30^^,^^31^ Thanks to this role in plant nutrition, there is increasing interest in application or promotion of AM fungi in food production.^32^

By improving crop access to soil nutrients, AM fungi have the potential to reduce the need for application of artificial fertilisers^33^ the over application of which can have detrimental effects on crop growth, the wider environment, and to human health.^34^ As such, reducing the necessity of using these synthetic amendments is a particularly appealing prospect for urban horticultural growers who often use fewer chemical interventions in management of their plots than commercial growers.^6^ However, the accumulation of heavy metals in urban soils^9^ reduces the abundance and diversity of microorganism species in the soil, including beneficial AM fungi, leading to a potential conflict in terms of desired versus realized outcomes for growers. In particular, the impact of heavy metal accumulation in urban soils on the function of AM symbioses is unknown, representing an important knowledge gap and barrier to their deployment in sustainable urban horticultural systems.

To date, research focus on the impact of soil-borne Zn on food production has been centered primarily on alleviating Zn deficiency in agricultural systems^35^ or on the impacts of Zn toxicity on both plants and soil microbes in sites which have extreme levels of contamination. These include sites such as mining and tailing sites^36^^,^^37^ on which it would not be feasible to produce food. However, the current drive to expand food production in cities necessitates an understanding of the impact of Zn at the moderate and high levels prevalent in soils used for urban horticulture.^9^

To provide new insights into the functional significance and potential for exploitation of AM fungi in urban horticulture, we investigated the impacts of Zn on the function of AM fungi in a crop commonly grown^38^ in urban horticultural scenarios. Given that high concentrations of Zn in soil retard AM fungal germination, hyphal extension, and colonization of host plant roots,^39^^,^^40^ we expect there to be a reduction in the nutritional benefits to host plants derived from AM colonization in Zn contaminated soils compared to in uncontaminated soils. We tracked the bi-directional exchange of plant-fixed carbon for AM fungal acquired P and N using radio- and stable isotope tracers in plants grown in soils contaminated with an urban environmentally relevant gradient of Zn.

## Results

### Plant growth

**Figure 1:**
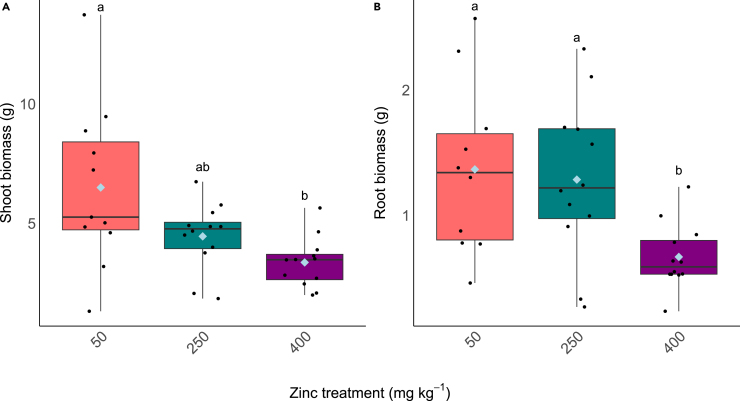
Biomass of freeze-dried plant tissue (A) Shoot biomass, (B) root biomass. Boxplots extend from the first to the third quartiles with the horizontal line signifying the median and the whiskers extending to the maximum and minimum data points. The mean is represented by the blue diamond and the individual data points are shown as black circles. Different letters denote significance difference (*p* < 0.05, one-way ANOVA, Tukey HSD).

Total plant biomass was less in plants grown at 400 mg kg^−1^ compared to those exposed to the lower concentrations of Zn in the soil (Figure 1). Dry root biomass was significantly lower in 400 mg kg^−1^ Zn compared to plants grown at 50 or 250 mg kg^−1^ Zn (Figure 1A, F_(2,32)_ = 4.835, *p* < 0.05) and shoot biomass was significantly less when grown in the 400 and 250 mg kg^−1^ Zn soils compared to the 50 mg kg^−1^ Zn treatment (Figure 1B, F_(2,32)_ = 4.17, *p* < 0.05).

### Root colonization by AM fungi

**Figure 2:**
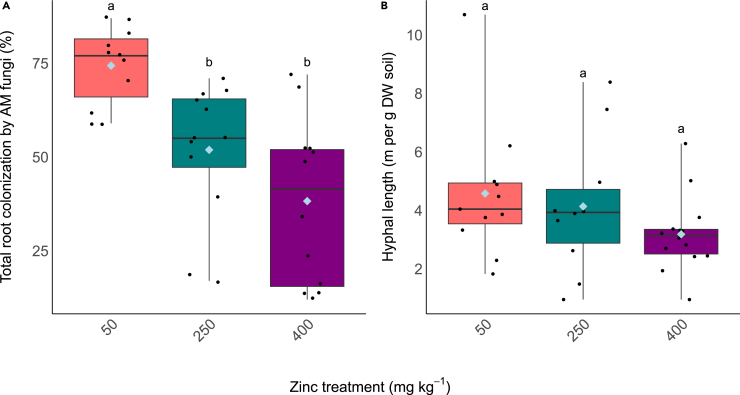
The impact of different Zn soil concentrations on AM colonization of the roots (A) Total percentage root length colonization, (B) extraradical hyphal length in the soil. Boxplots extend from the first to the third quartiles with the horizontal line signifying the median and the whiskers extending to the maximum and minimum data points. The mean is represented by the blue diamond and the individual data points are shown as black circles. Different letters denote significance (*p* < 0.05, (A) Kruskal-Wallis, Dunn’s test, (B) one-way ANOVA, Tukey HSD).

All plants in all Zn treatments were colonized by AM fungi (Figure S1). The percentage root colonization decreased with increasing soil Zn concentrations, with highest colonization (%) in plants grown in 50 mg kg Zn and incrementally less in those grown in 250 mg kg^−1^ and 400 mg kg^−1^ (Figure 2A H_(2)_ = 16.902; *p* < 0.05). Extraradical hyphal lengths in the soil did not significantly differ across the different Zn treatments.

### Mycorrhizal and plant-acquired phosphorus content of plant shoots

**Figure 3:**
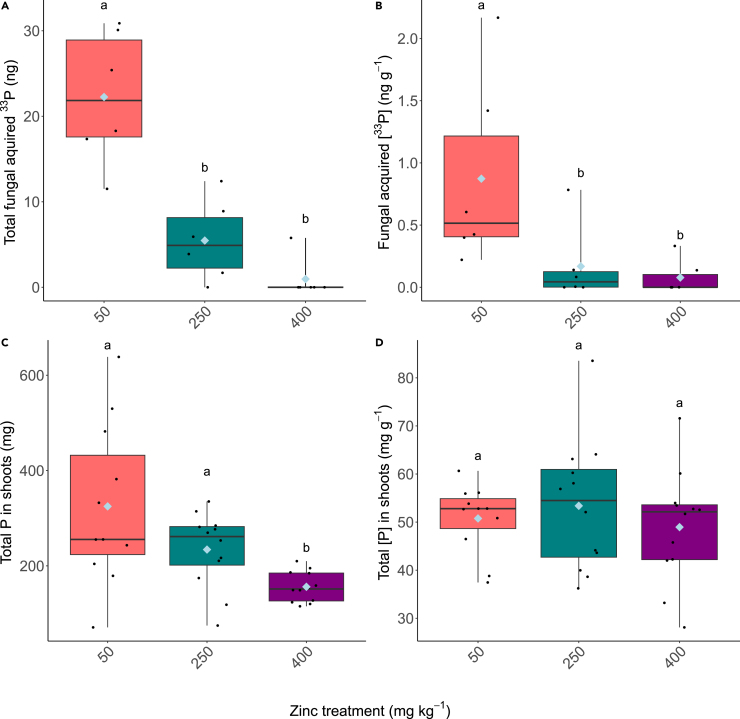
Phosphorus contents in the plant shoots (A) Total fungal acquired ^33^P, (B) amount of fungal acquired ^33^P per g of shoot tissue, (C) total amount of P (^33^P+^31^P) detected in the shoot tissue, (D) total P (^33^P+^31^P) detected per gram of shoot tissue. Boxplots extend from the first to the third quartiles with the horizontal line signifying the median and the whiskers extending to the maximum and minimum data points. The mean is represented by the blue diamond and the individual data points are shown as black circles. Different letters denote significance (*p* < 0.05, Kruskal-Wallis, Dunn’s test).

There were significantly lower amounts of AM fungal acquired ^33^P detected in the plant host shoots in the 250 mg kg^−1^ and 400 mg kg^−1^ Zn treatments compared to the lower soil Zn treatment with the shoots of plants grown at 50 mg kg^−1^ possessing a much greater amount of fungal acquired ^33^P than those plants grown in higher Zn concentrations (Figure 3A; H_(2)_ = 13.199, *p* < 0.05. Figure 3B; H_(2)_ = 9.2584, *p* < 0.05). Most plants grown at the highest Zn treatment contained no detectable ^33^P. This is in contrast to the concentration of P (plant and fungal acquired) within the plant shoots which was not significantly different between Zn treatments (Figure 3C; F _(2,32)_ = 0.465, *p* = 0.632), however the total P per plant was significantly reduced in plants grown at 400 mg kg^−1^, in line with the observed reduction in shoot biomass (Figure 3D; H_(2)_ = 11.338, *p* < 0.05).

### Plant to AM fungi C transfer

**Figure 4:**
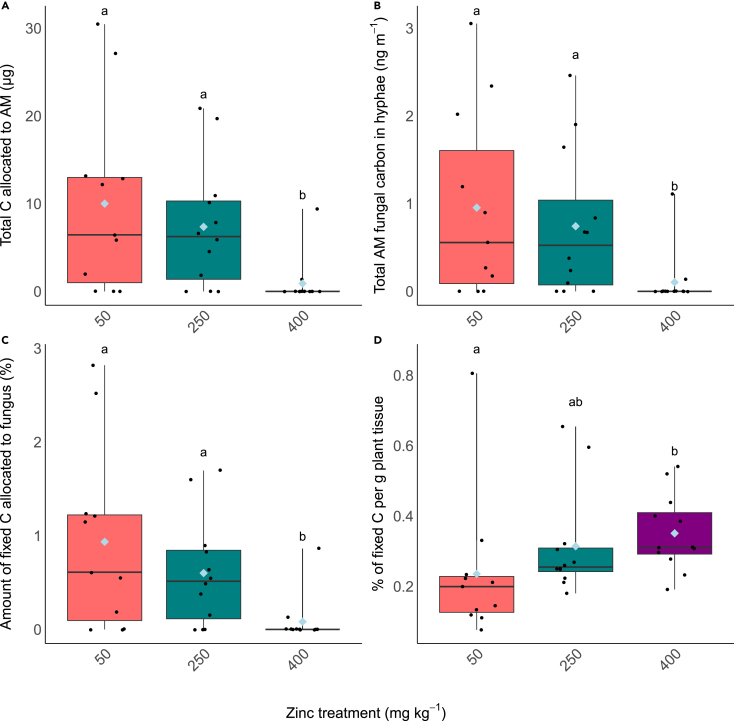
Carbon content in the static cores (A) Total carbon (^12^C+^14^C) allocated by the plant to the fungus, (B) the total C detected per g of soil, (C) the percentage of C fixed by the plant and transferred to the fungus and (D) the percentage of C fixed by the plant per g of plant tissue. Boxplots extend from the first to the third quartiles with the horizontal line signifying the median and the whiskers extending to the maximum and minimum data points. The mean is represented by the blue diamond and the individual data points are shown as black circles. Different letters denote significance, (A, B and C *p* < 0.05, Kruskal-Wallis, Dunn’s test, D, one-way ANOVA, Tukey’s HSD).

Plants grown with 50 mg kg^−1^ Zn fixed significantly less C relative to their biomass compared to plants grown at 400 mg kg^−1^ (Figure S1, H_(2)_ = 10.321, *p* < 0.05). Despite this, the total allocation of plant-fixed carbon to the fungus was significantly reduced at the highest Zn treatment (Figure 4A; H_(2)_ = 10.438, *p* < 0.05), whereas there was no difference in the amount of carbon allocated to AM fungi in pots treated with 50 or 250 mg kg^−1^. The concentration of C within fungal hyphae was likewise significantly reduced in the highest Zn treatment (Figure 4B; H_(2)_ = 9.707, *p* < 0.05),. The percentage of recently fixed C in the fungus also decreased with increasing Zn from c. 0.94%–0.60% and 0.08% at 50 mg kg^−1^, 250 mg kg^−1^ and 400 mg kg^−1^ respectively (Figure 4C; F_(2,32)_ = 4.75, *p* < 0.05, *p* < 0.05) with the majority of the plants grown at 400 mg kg^−1^ transferring no carbon to the AM. The plants grown at 400 mg kg^−1^ Zn fixed more carbon per g of plant tissue (Figure 4D; F(_2,32)_, *p* < 0.05).

### Assimilation of zinc into plant and association with fungal biomass

**Figure 5:**
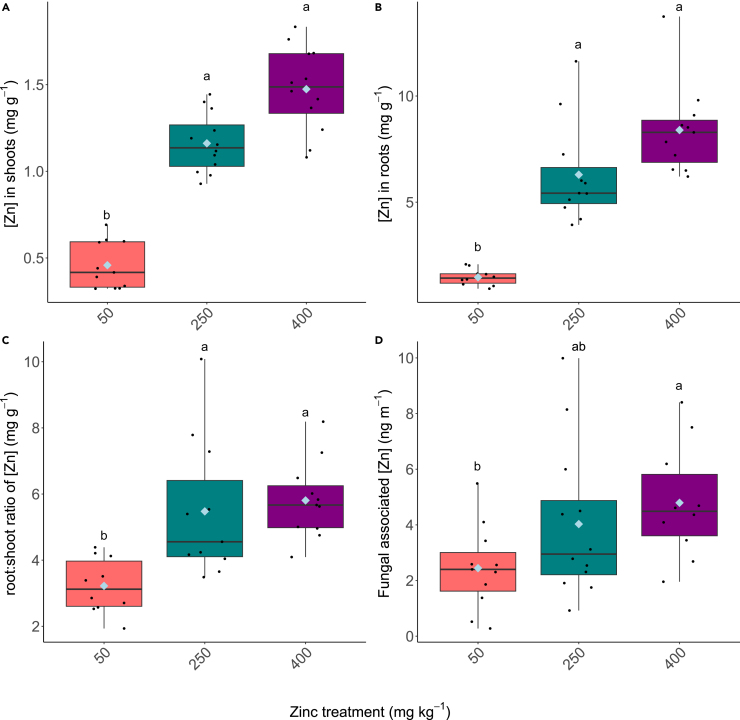
Zinc content of the plant and fungal materials (A) Total Zn in the shoots (B) total Zn in the roots, (C) the ratio between the concentration of Zn in the roots and the shoots (D) the concentration of fungal associated Zn per meter of hyphae. Boxplots extend from the first to the third quartiles with the horizontal line signifying the median and the whiskers extending to the maximum and minimum data points. The mean is represented by the blue diamond and the individual data points are shown as black circles. Different letters denote significance (*p* < 0.05, Kruskal-Wallis, Dunn’s test).

The amount of Zn assimilated into the aboveground plant tissues significantly increased at 250 mg kg^−1^ and 400 mg kg^−1^ compared to 50 mg kg^−1^ (Figure 5A; H_(2)_ = 26.929, *p* < 0.05). This pattern was also observed in the pea roots (Figure 5B; H_(2)_ = 22.905, *p* < 0.05) alongside a greater ratio of Zn detected in the roots compared to in the shoots roots (Figure 5C; H_(2)_ = 15.746, *p* < 0.05). A significantly greater amount of Zn was found associated with the extraradical hyphal biomass extracted from pots containing 400 mg kg^−1^ Zn than in the other Zn treatments (Figure 5D; H_(2)_ = 6.9942, *p* = 0.03).

## Discussion

Many land management practices such as the applications of manures and fertilizers to agricultural land can increase the concentration of Zn in the soil, potentially reducing the productiveness of urban horticultural soil at a time when food production needs to be increased to meet a growing population. Soil microbes, such as AM fungi, have the potential to help sustainably increase food production by improving crop access to soil nutrients^29^^,^^30^ and enhancing resistance to pests and pathogens,^41^^,^^42^^,^^43^ reducing the need for application of fertilizers and pesticides. However, to date, there has been very little research carried out on how the accumulation of contaminants in the soil environment impacts the functionality of AM fungi. Here, we investigated the impact of increasing, environmentally-relevant, concentrations of Zn in the soil impacts AM symbioses in an important horticultural crop frequently grown in urban soils.

### AM colonization of plant roots is negatively impacted by soil zinc contamination

Colonization of the pea roots by AM fungi was impacted by increased concentration of Zn in the soil. This is in line with previous studies which also report a decrease in mycorrhizal colonization in response to exposure to Zn in the soil, albeit at lower concentrations than were used in this investigation,^44^ although others have shown a decrease^45^^,^^46^ or no effect^47^ at similar concentrations to those used here. There is likely to be an effect of soil type, plant and fungal species, and growth conditions^48^^,^^49^ on these observations, although the mechanisms underpinning the interaction between Zn concentration in the soil and the extent of AM colonization remain unclear. Despite the decrease in root colonization, the density of extraradical fungal hyphal mycelium in the soil of pots containing higher levels of Zn was similar to those exposed to 50 mg kg^−1^ Zn suggesting that the increase in Zn may not be directly toxic to the fungus and that the scavenging potential of the AM fungal hyphae may not be impacted at these concentrations. Although there was a reduction in colonization at 250 mg kg^−1^, the biomass of the plant was only significantly affected at the highest Zn treatment which resulted in both a lower shoot and root biomass.^18^ There are contrasting reports of there being a Zn fertilization effect in terms of shoot biomass, with increases^50^ and lack of response^51^ being reported in different crops.^50^^,^^51^ However, at elevated concentrations, Zn has been shown to consistently negtively impact plant biomass.^18^^,^^47^^,^^52^^,^^53^ This phenotype may be due to oxidative stress inhibiting growth through the disruption of processes such as photosynthesis,^18^ although in our experiment this likely wasn’t the case as plants in the lowest Zn treatment fixed the most C during the labeling period compared to those in the higher Zn treatments. Our results suggest that reduced colonization by AM fungi is likely to be mediated by the plant rather than there being any direct effect on the AMF. It is important to note that the extent of root colonization observed through staining is unlikely to be directly representative of the functionality of AM fungi *in planta* as the stain will bind to both live and dead fungal structures whether nutrient exchange is occurring or not.

Contamination of the soil with the higher concentrations of Zn in our experiments drove reduction – and even cessation – of mycorrhizal ^33^P transfer to host plants, regardless of the amount of colonization present in the host plant root system. This suggests there may be alternative benefits for host plants in maintaining associations with AM fungi when soil is contaminated with Zn. These could include increased activity of antioxidative enzymes to reduce the impact of reactive oxygen species or increased rates of photosynthesis.^16^^,^^54^^,^^55^ Plants grown in 250 mg kg^−1^ Zn soil achieved a similar biomass at harvest as the plants grown in the lower Zn concentration, which suggests that there may be a role for AM associations beyond P transfer, particularly as hyphal density in the soil was maintained across Zn treatments. It is possible that increased fungal biomass within the pot may have a protective effect through the increased production of glomalin-related soil protein which is exuded by AM fungal hyphae^56^^,^^57^ and may bind and sequester heavy metals in soil,^58^ including Zn.^59^^,^^60^^,^^61^^,^^62^ Alternatively, other studies have shown that AMF hyphae themselves store and sequester heavy metals,^63^ thereby preventing them from being transported to the plant tissues.

### High concentration of zinc in soil impacts AM transfer of ^33^P to plant host

Despite the reduction in P supplied by AMF to plants treated with 250 mg kg^−1^ and 400 mg kg^−1^ Zn, the plants in our experiments still acquired P from the soil, either directly or indirectly via alternative microbial nutrient cycling processes, largely maintaining the concentration of P in their tissues. However, plants grown in 400 mg kg^−1^ Zn treated soil had a lower biomass at harvest compared to the low Zn treatment. 250 mg kg^−1^ is the median value for Zn content in urban horticultural soils across the UK^9^ suggesting that crops grown in soils where Zn levels are elevated may benefit less from AM symbiosis with regards to nutrient uptake than in soils with lower Zn content. Additionally, despite the large reduction in fungal acquired P, the total amount of P detected in the plant shoots was maintained, indicating that plants in high Zn treated soils may have switched their strategy for the acquisition of P from the soil, thereby apparently reducing reliance on AMF for uptake of nutrients. This is likely to be important in horticultural scenarios where plant-available soil P may become increasingly depleted, necessitating greater application of P fertilizers.

The mechanisms and drivers for the reduction in AM ^33^P transfer to host plants in our experiments are not yet clear. It may be that Zn in soil is toxic to AM fungi and so fungal hyphae growing out of colonized roots are growing away from the plant to reach a region within the soil lower in Zn concentration, allowing spores that are produced to have a greater chance of successful germination and colonization of plants in the future. In this scenario, the fungi may transfer less P to the host plant because the fungi place most of their resources into growth rather than incur the cost of actively producing phosphatases and transporters to take up and translocate P to the plant. It is possible that there are interactions between Zn and AM P transporters on the plant and/or fungal symbiotic interfaces. Several antagonistic and complementary interactions between the uptake of these two elements have previously been described, with high levels of Zn being shown to decrease shoot to root translocation in rice plants.^64^ It is therefore possible that a similar response is present in the translocation of P along hyphae due to the phylogenetic similarities between plant and fungal P transporters.^65^ There may also be alternative interactions occurring, both in the soil and within the plant and fungus, between these two elements and other nutrients or soil components that impact on the uptake of P, particularly in urban soils where land management techniques and industrial legacy have led to distinct soil characteristics such as increased black carbon and other heavy metals.^9^^,^^11^ When assessing the impacts of different factors on mycorrhizal symbioses, the vast majority of published studies use colonization as a benchmark to assess the status of the relationship. However, our results provide another clear demonstration that root colonization does not directly relate to the functionality of the symbiosis and highlights the need for further research on how the role of AM fungi may change under different environmental conditions.

### Transfer of carbon to fungal symbionts is reduced by increasing zinc soil concentrations

Despite the rate C fixation over the 24-h labeling period being greater when normalized to biomass in the plants grown at 400 mg kg^−1^, the percentage of recent photosynthates transferred to the AM fungus was significantly reduced in the highest Zn treatment of our experiments. Plants may discriminate and reduce C supply to their fungal partners when less P is transferred to the plant,^66^ although this relationship may be disrupted by different environmental conditions and stresses,^67^^,^^68^^,^^69^ We found that although the total amount of carbon fixed in the plants was increased at lower Zn levels, when normalized to biomass the concentration of carbon in the plant tissues was actually lower at 50 mg kg^−1^. Despite this, plants grown in soil Zn concentrations of 50 mg kg^−1^ and 250 mg kg^−1^ continued to invest carbon into AM fungi. Even when the assimilation of ^33^P by plants grown at 250 mg kg^−1^ was drastically reduced, the amount of carbon transferred to the fungus was unchanged. This may indicate that the fungus has become somewhat parasitic, drawing more resource from its host than imparting benefits. More likely, the AM fungi may be providing an alternative benefit for the host plant (e.g., sequestration of Zn in fungal tissues or promotion of plant tolerance/resilience to Zn), justifying continued carbon investment by the plant.

Partnerships with AM fungi are not only associated with improved nutrition for the host plant – the presence of AM fungi can provide increased resilience to a variety of biotic and abiotic stress factors. These include exposure to heavy metal contamination, with resilience being achieved through increased activity of antioxidative enzymes and/or increased photosynthetic rate.^54^^,^^55^ It is possible that, in our experiments, by investing carbon resources into the fungus (Figure 4), the plant host gains these advantages under stress but does not derive apparent nutritional benefit. The ‘dilution effect’ describes increased resilience of plants to heavy metals as being due to increased plant biomass facilitated by AM fungi, which, despite maintained uptake, results in a lower concentration of the contaminant metal in the plant tissues, and therefore reduces negative impacts on plant health.^70^ However, the increase in plant biomass in these cases is thought to be mediated by an increase in nutrient uptake via the AM fungi which is contrary to what we see in our experiments. Alternatively, AM induced increases in plant photosynthesis resulting in more carbon being fixed could in turn be directed toward plant growth.^71^ Our finding that while AM-acquired ^33^P was lower in plants exposed to high Zn contamination, the total P in plant tissues was not affected, suggesting that the plant ameliorated loss of AM-acquired P either through direct uptake via the roots, or via alternative microbial P cycling processes that liberate otherwise plant-inaccessible P sources in the soil.^72^ Previous studies have shown increasing Zn having a detrimental impact on shoot P although in these studies the presence of AM still provided a beneficial effect on shoot P compared to non-mycorrhizal plants.^73^^,^^74^

The Zn content of both shoots and roots increased with the higher Zn treatments compared to the lowest Zn concentration, although from our experiments it is not clear as to whether the transport of Zn into plant tissues was mediated by the plant roots or the fungus. Previous studies suggest that AM fungi transport Zn to plant host tissues from the soil^31^ and, in doing so, can contribute to Zn accumulation in cereal crops^51^ with results suggesting that AM fungi are unable to limit the amount of Zn transported^51^ This may have important implications for growing crops in soils where Zn is often present in excess, such as those used for urban horticulture. In our experiments, significantly more Zn was detected in the hyphal extractions at 400 mg kg^−1^ than in the other Zn treatments, although it is not certain that this Zn is bound within the hyphal biomass itself or whether it is more loosely associated with the hyphal mycelium. Regardless, Zn can be translocated by fungal hyphae,^31^ therefore it is possible that the increase in Zn found within the plant tissues in our experiments is at least partially due to direct transport by the fungi. Alternatively, AM fungi are able to sequester other heavy metals within the fungal structures^54^^,^^70^ and thus it is likely that at least some of the Zn measured within root tissues reflects Zn bound up within the fungal cell walls as well as that within the plant root tissues. The ratio of Zn in the roots compared to the shoots was significantly higher in plants grown at 250 and 400 mg kg^−1^ compared to the lower Zn treatment, suggesting that Zn may increasingly be sequestered in the roots at higher concentrations as observed previously in AM colonized plants exposed to other heavy metals.^75^ It may be that Zn is preferentially sequestered in the intraradical fungal structures themselves, however there is evidence to suggest that mycorrhizal root cell walls undergo structural changes,^70^ thereby facilitating the binding of Zn in the roots and preventing translocation to aerial tissues. Finally, as previously mentioned, GRSP may be exuded by the fungus and could be associated with the external surfaces of the extraradical hyphae. Consequentially a fraction of the detected Zn in the hyphal extractions could be bound to this substance.

Further research is needed to establish whether the community composition of AM fungi is altered by exposure to elevated Zn concentration in the soil, particularly in the context of urban horticulture. It is important to note that fungal species that are present in urban settings may have adapted to the soil conditions and consequentially may offer a greater functionality when in symbiosis with compatible plant hosts. It has been observed that strains indigenous to contaminated sites tend to have a level of adaption to heavy metal contamination^76^^,^^77^ although this tolerance appears to be rapidly reduced if isolated strains are cultivated without the presence of heavy metals.^78^ This observation may be relevant in the context of ongoing horticultural practices which continue to add excess amounts of Zn to the soil through various amendments such as manure, where it occurs at a rate faster than the microbial communities can adapt. Severely contaminated sites show large declines in the diversity of fungal species^79^^,^^80^ and therefore there is a risk of disrupting the fungal communities in urban soils if Zn continues to accumulate. Additionally, it is important to note that Zn is not the only contaminant present and that there are lots of other confounding variables such as the presence of other heavy metals and other substances which have been found to be elevated in urban soils, such as black carbon^9^ which will likely alter the availability of the Zn and perhaps mitigate its more toxic effects. Other soil pollutants, e.g., pharmaceutical antifungals^81^ and chemical pesticides,^82^ have also been shown to lessen the amount of P acquired by the fungus indicating that the functionality, and yield benefits derived thereof, of AM symbioses may be considerably affected by anthropogenic pollution.

With the pressing need to produce more food more sustainably and securely for a growing global population, urban areas could provide much needed productive land. However, due to the distinct properties often found in these soils, in particular excessive heavy metal contamination such as Zn, further work is needed to advance our understanding on how these different factors may affect the fungal communities present individually and in combination, and how this in turn may affect crop growth, yields and nutrition. Our observation of disruption in carbon and nutrient transfers between AM fungi and crops emphasizes the intricate interplay between soil health, heavy metal contamination, and urban horticultural management practices. To achieve resilient and sustainable urban horticulture, a nuanced understanding of the consequences of soil contamination, such as elevated zinc concentrations, on soil functionality is critical. Our findings emphasize the need for targeted interventions and regulatory measures to mitigate the adverse effects of heavy metal pollutants, ensuring the long-term viability of urban soils for food production and contribution to global food security goals.

### Limitations of the study

The results of this study show that increasing levels of zinc have a detrimental impact on the nutrient exchange between symbiotic partners. In field conditions this effect may be modulated by other factors such as additional heavy metals or other soil properties. Additionally, the means by which the soil was spiked may have resulted in different a different bioavailability of Zn than in ‘real soils’ although the mechanisms behind availability of heavy metals and other nutrients in the soil are poorly understood. Finally, our method for extraction and analysis of Zn in fungal hyphae does not allow us to determine whether Zn detected in our fungal biomass samples is representative of the Zn within the hyphae, whether it is bound externally to the fungal structures, or whether it is run off from the soil sample itself.

## STAR★Methods

### Key resources table

**Table:** 

REAGENT or RESOURCE	SOURCE	IDENTIFIER
**Chemicals, peptides, and recombinant proteins**		

Acetic acid - glacial	VWR	8187552500
^33^P-phosphoric acid	Hartmann Analytic	FF-01
Lactic acid 90%	Acros	189870010
^14^C-sodium bicarbonate	Revvity	NEC086H001MC
Potassium Hydroxide	Acros	134060010
Ethanol	Sigma	32221
Pelikan Brilliant Black ink	NA	NA
Poly(vinyl alcohol)	Sigma	363146
Glycerol	Acros	158920025
Sulfuric acid	VWR	20700.323
Emulsify-safe	Revvity	6013389
Ammonium Molybdate.4H_2_O	Generon	AB0067
Ascorbic acid	Sigma	A92902
CarbonCount	Meridian Biotechnologies	CC/10
CarbonTrap	Meridian Biotechnologies	CT/10
Hydrogen Peroxide 35%	Acros	202460010
Sucrose	ThermoScientific	A15583

**Experimental models: Organisms/strains**		

*Pisum sativum* cv. Meteor	Premier Seeds Direct	PEA10
Mycorrhizal innoculum	PlantWorks, UK	N/A

**Software and algorithms**		

R v4.2.1		https://rstudio.com
R Studio 2022.07.01		RStudio, Inc

### Resource availability

#### Lead contact

Further information and requests for resources should be directed to the lead contact Katie Field k.j.field@sheffield.ac.uk.

#### Materials availability

This paper does not report original code.

This study did not generate unique reagents.

#### Data and code availability

Any additional information required to reanalyse the data reported in this paper is available from the lead contact upon request.

### Method details

#### Experimental design

A 1:1 mix of sand and topsoil v/v twice autoclaved at 124°C for 30 min, before being dried at 70°C. The soil was then spiked with a 0.5M solution of Zn sulfate (ZnSO_4_)^44^ and left to fully dry for one week. ZnSO_4_ has been commonly used in similar soil spiking experiments^45^^,^^83^^,^^84^^,^^85^ and additionally is often used both as a livestock food supplement and as a chemical fertiliser for food production.^50^^,^^86^ The spiked soil was mixed into pots together with 35 g of commercially available mycorrhizal inoculum (PlantWorks, Rootgrow Professional, UK) containing the AM fungal species *Funneliformis mosseae, Funneliformis geosporus, Claroideoglomus clarodeum, Rhizophagus intraradices, Glomus micoraggregarum, Diversispora* spp, in a clay particulate carrier matrix, so that there were 12 individual replicates of each of three Zn soil concentrations of 50 mg kg^−1^, 250 mg kg^−1^ and 400 mg kg^−1^. The concentrations used are relevant to Zn levels found in urban horticultural soils) with 363.3 mg kg^−1^ being the median value for one city, 250 mg kg^−1^ being the UK median value, sampled and a national range of 46.16–1213.0 mg kg^−1 9^.

Pea (*Pisum sativum*) seeds (cv. Meteor, Premier Seeds Direct, UK) were sown in a hole containing an additional 15 g inoculum to ensure immediate contact between the AMF propagules and developing roots. Given the near-ubiquity of AM fungi in urban soils,^28^^,^^87^ a scenario where a receptive plant wouldn’t be colonised by AM fungi would be atypical. Because of this, we do not include a non-mycorrhizal control in our experiments. Instead, two windowed cores were inserted into the soil in each pot. The windows of the cores were covered with a 35μm nylon mesh (Plastok, UK) which is fine enough to exclude root growth but allows fungal hyphal access to the interior of the core.^88^ Cores were filled with soil from the pot at the time of planting. Upon commencement of isotope tracing (see below), one core in each pot was rotated to sever AM fungal connections between the core contents and the host plant, providing internal controls for relative contribution of alternative microbial cycling and passive diffusive processes to plant nutrition in each pot (more details below).

#### Plant growth conditions

Plants were grown in a randomised layout on raised benches within a controlled environment glasshouse with a temperature of 18°C and a night temperature of 16°C. Natural light was supplemented with LED lighting to give 16 h of illumination (simulating natural daylight) each day. Pots were rotated randomly around the glasshouse once a week and watered as needed.

#### Radioisotope tracing of carbon for nutrient exchange between plants and AM fungi

At 7 weeks 100 μL solution containing 1 Mbq ^33^P-orthophosphate (111 TBq mmol^−1^ specific activity, [^33^P]-phosphoric acid; Hartmann Analytic, Braunschweig, Germany) was added to one of the cores in each pot. To the other core 100 μL of distilled water (dH_2_O) was added. In half the pots the ^33^P-labelled cores were rotated every 48 h to sever any hyphal connections that had formed between the plant roots and the core interior whereas for the other half the ^33^P-labelled cores were kept static to allow the hyphae to proliferate in the core interior.

Three weeks after application of the ^33^P labeling solution, the shoots of the plants were enclosed and sealed in labeling chambers to create an airtight headspace around the plant. At the beginning of the photoperiod, 2 mL 80% lactic acid was added to a square cuvette containing 27 μL ^14^C-sodium bicarbonate (1.62GBq mmol^−1^ specific activity), liberating 1 MBq ^14^CO_2_ into the headspace.

#### Plant harvest

After 24h, 4 mL of 2M potassium hydroxide (KOH) was injected into vials placed in the headspace to capture any remaining gaseous ^14^CO_2_ before bags were opened. Cores were removed from the soil and the shoots separated from the roots. Roots were cleaned in tap water and a subset of approximately 0.2g (FW) was sampled for clearing and staining (see below) to quantify the extent of mycorrhizal colonisation. In addition to the soil within the static and rotated cores, a sample of bulk soil was also taken and frozen at −80°C for extraction of fungal hyphae. Roots, shoots and the soils taken from the cores were freeze dried before being weighed prior to further analysis. Additionally, any root nodules that had formed over the course of the experiment were removed and weighed.

#### Colonisation of plant roots by AM fungi

Fresh root samples were placed in 10% KOH (w/v) and heated at 60°C for 45 min to break down organelles in the plant root cells. An ink and vinegar solution (5% Pelikan Brilliant Black, 5% acetic acid, 90% dH2O) was then used to stain the fungal structures in the root overnight before de-staining in 1% acetic acid. Polyvinyl lacto-glycerol (PVLG) was used to mount the roots on microscope slides which were then observed at 100× magnification to assess percentage root length colonisation.^89^

AM hyphae were extracted from 3 g bulk soil by mixing in 700 mL water. A 200 mL subsample was then decanted into a separate beaker, stirred for 30 s before 10 mL was extracted and filtered through 45 μm membrane filters in order to collect the hyphae on the membrane surface. Ink and vinegar stain was then added dropwise to the filter followed by dH_2_O to wash away excess. The filter circles were mounted on microscope slides using PVLG and observed at 100× magnification. AM fungal hyphal lengths were calculated using the gridline-intersection methodology^89^ with 100 fields of view.

#### Quantification of fungal acquired ^33^P and total P

Approximately 30 mg of homogenised freeze-dried shoot material was taken in duplicate and digested in 1 mL concentrated sulfuric acid overnight. Samples were then heated to 350°C for 15 min before being allowed to cool, at which point 100 μL chilled hydrogen peroxide was added to each tube and heated again for 1 min in order to decompose any remaining organic residues. Samples were diluted to 10 mL with dH_2_O and a 2 mL aliquot was added to 10 mL of liquid scintillant (Emulsify-safe, PerkinElmer) for quantification of radioactivity through liquid scintillation (TriCarb 4910TR, PerkinElmer). The amount of ^33^P in each sample was calculated using published equations (see supplemental information;^90^) the mean ^33^P content detected in plant tissues where the labeling solution was added to the rotated core was subtracted from each value for ^33^P in plant tissues for plants grown in pots where hyphae had access to the labeling solution (static cores) within each Zn treatment. This step accounts for non-mycorrhizal mediated transfer of ^33^P by processes out of the core such as diffusion or alternative microbial cycling.

Total P content in the shoot was ascertained by adding 0.5 mL of the digest sample to a cuvette containing 0.5 mL ammonium molybdate and 0.4 mL ascorbic acid solutions were made up to 3.8 mL using dH_2_O. A 10 mg ml^−1.^ P solution was used to make up cuvettes with known amounts of P in order to generate a standard curve from which the mass of total P in the samples could be calculated. Solutions were left for 45 min before measuring optical density at 882 nm using a spectrophotometer (Jenway 6300).

#### Quantification of plant to AM fungi C transfer

Approximately 30 mg of freeze-dried soil from the static and rotated cores was oxidised (Model A307 Sample Oxidiser, PerkinElmer) to release any fixed ^14^C as ^14^CO_2_ which was subsequently captured in 10 mL CarbonTrap (Meridian Biotechnologies Ltd, Tadworth, UK) and mixed with 10 mL CarbonCount (Meridian Biotechnologies Ltd, Tadworth, UK). Radioactivity was then quantified by liquid scintillation and the values used to calculate the amount of ^12^C also transferred using published equations (see supplemental information)^91^ The total C (^14^C + ^12^C) fixed by the plant and transferred to the fungus was calculated by subtracting the total C value for the rotated core by that of the static core.

#### Zinc quantification

Plant tissue was ball-milled (Tissuelyser II, Qiagen) to create a fine powder and pressed into a pellet using a manual hydraulic press (Specac, Orpington, UK) at 11 tons for 2 s. A commercial P-XRF (portable X-ray fluorescence) instrument (Niton XL3t900 GOLDD Analyzer; Thermo Scientific, Winchester, UK) was used to analyze the concentration of Zn in the different plant tissues.^92^ To separate hyphae from soil, bulk soil was dispersed in 30mL ultra-pure water before being centrifuged at 3100 x g for 3 min. The supernatant was discarded, and the pellet thoroughly resuspended in 50 mL 45%(w/v) sucrose solution. Samples were then centrifuged at 50 x *g* for one minute.

The supernatant was then washed through a 30 μm nylon mesh which was then in turn washed into tubes with 1.5mL ultrapure water (modified from Awad and Pena, 2023; Bingle and Paul, 1986).^93^^,^^94^ Samples were freeze dried before being digested in aqua regia. Zn levels were analyzed by ICP-MS (Agilent 7500ce).

#### Statistical analyses

Statistical analyses were performed in RStudio (R Studio 2022.07.01). Q-Q plots and Shapiro-Wilk tests were used to assess the normality of the datasets after log transforming the data. Where data was found to fit assumptions of normality after log transformation, one-way ANOVA tests were performed followed by post-hoc Tukey HSD tests to assess significance between treatments. For datasets which could not be normalised a Kruskal-Wallis test was used in analysis followed by Dunn’s post-hoc test.
