# Supplementary material for: Impact of zinc on arbuscular mycorrhizal-mediated nutrient acquisition in urban horticulture
Source: iScience. 2024 Jul 25;27(8):110580. doi: 10.1016/j.isci.2024.110580 (PMC11363573; doi:10.1016/j.isci.2024.110580)
Supplement: Document S1. Figure S1 and Methods S1–S3 [file mmc1.pdf]

iScience, Volume *27*

## Supplemental information

# Impact of zinc on arbuscular mycorrhizal-mediated nutrient acquisition in urban horticulture

**Miles P.A. Bate-Weldon, Jill L. Edmondson, and Katie J. Field**

## Supplementary file

**Figure S1 – A micrograph showing the presence of AM within the roots of pea plants grown at (A) 50 mg $kg^{-1}$, (B) 250 mg $kg^{-1}$and (C) 400 mg $kg^{-1}$ at 10x magnification, related to Figure 2**

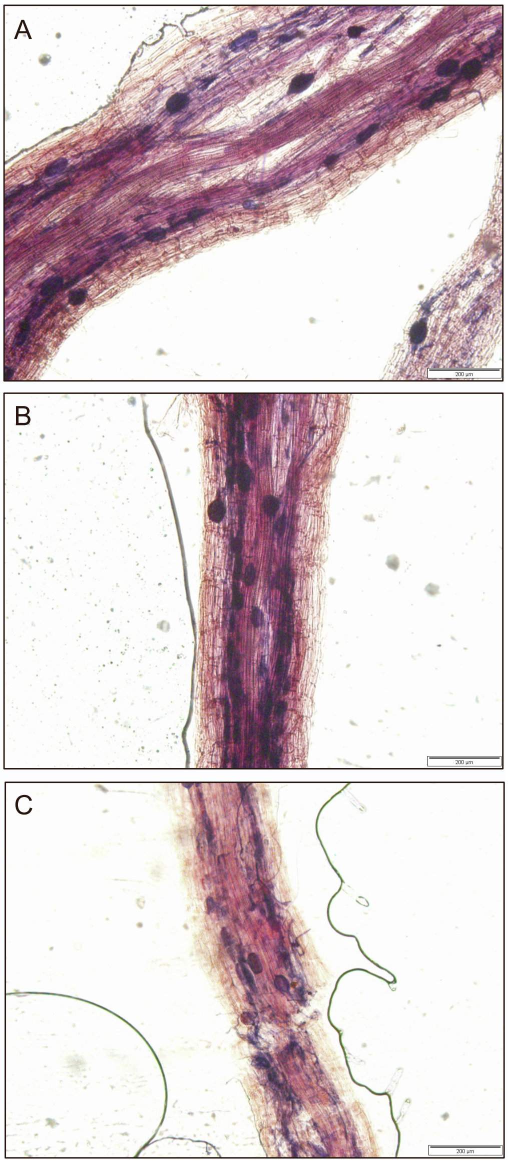

**Methods S1 - An equation for calculating the amount of $^{33}P$ transferred to the plant via the fungus in the core, related to Figure 3**

$$m_{33P} = \left\{\left[\frac{C_{DPM}/60}{SpACT}\right] M_{wt(33P)}\right\} D_f$$

*$m_{33P}$, mass of $^{33}P$ (mg); $C_{DPM}$, counts as disintegrations per minute; SpACT, specific activity of the isotope (GBq $mmol^{-1}$), percentage of plant fixed carbon supplied to the fungus; $M_{wt(33P)}$, molecular weight of $^{33}P$, $D_f$; dilution factor.*

**Methods S2 - An equation for calculating the amount of $^{14}C$ transferred to the fungus in the core, related to Figure 4**

$$m_{14C} = \left\{\frac{\left[\frac{C_{DPM}/60}{10^9}\right]}{SpACT}\right\} M_{wt(14C)}$$

*$m_{14C}$, mass of $^{14}C$ (mg); $C_{DPM}$, counts as disintegrations per minute; SpACT, specific activity of the isotope (GBq $mmol^{-1}$); $M_{wt(14C)}$, molecular weight of $^{14}C$.*

**Methods S3 - An equation for calculating the amount of $^{12}C$ transferred to the fungus in the core, related to Figure 4**

$$m_{12C} = \left\{0.2729 \left[\frac{A}{100}\right] M_{wt}\right\}$$

*$m_{12C}$, mass of $^{12}C$ (mg); 0.2729, the proportion of C in $CO_2$; A, percentage of plant fixed carbon supplied to the fungus; $m_{CO2}$, mass of $CO_2$ in the headspace.*

**Formatted:** Don't adjust space between Latin and Asian text, Don't adjust space between Asian text and numbers
